# Long-term results after Boston brace treatment in late-onset juvenile and adolescent idiopathic scoliosis

**DOI:** 10.1186/1748-7161-6-18

**Published:** 2011-08-31

**Authors:** Johan Emil Lange, Harald Steen, Ragnhild Gunderson, Jens Ivar Brox

**Affiliations:** 1 Orthopaedic and Radiological Department, Rikshospitalet, Oslo University Hospital, Norway

## Abstract

**Background:**

It is recommended that research in patients with idiopathic scoliosis should focus on short- and long-term patient-centred outcome. The aim of the present study was to evaluate outcome in patients with late-onset juvenile or adolescent idiopathic scoliosis 16 years or more after Boston brace treatment.

**Methods:**

272 (78%) of 360 patients, 251 (92%) women, responded to follow-up examination at a mean of 24.7 (range 16 - 32) years after Boston brace treatment. Fifty-eight (21%) patients had late-onset juvenile and 214 had adolescent idiopathic scoliosis. All patients had clinical and radiological examination and answered a standardised questionnaire including work status, demographics, General Function Score (GFS) (100 - worst possible) and Oswestry Disability Index (ODI) (100 - worst possible), EuroQol (EQ-5D) (1 - best possible), EQ-VAS (100 - best possible), and Scoliosis Research Society - 22 (SRS - 22) (5 - best possible).

**Results:**

The mean age at follow-up was 40.4 (31-48) years. The prebrace major curve was in average 33.2 (20 - 57)°. At weaning and at the last follow-up the corresponding values were 28.3 (1 - 58)° and 32.5 (7 - 80)°, respectively. Curve development was similar in patients with late-onset juvenile and adolescent start. The prebrace curve increased > 5° in 31% and decreased > 5° in 26%. Twenty-five patients had surgery. Those who did not attend follow-up (n = 88) had a lower mean curve at weaning: 25.4 (6-53)°. Work status was 76% full-time and 10% part-time. Eighty-seven percent had delivered a baby, 50% had pain in pregnancy. The mean (SD) GFS was 7.4 (10.8), ODI 9.3 (11.0), EQ-5D 0.82 (0.2), EQ-VAS 77.6 (17.8), SRS-22: pain 4.1 (0.8), mental health 4.1 (0.6), self-image 3.7 (0.7), function 4.0 (0.6), satisfaction with treatment 3.7 (1.0). Surgical patients had significantly reduced scores for SRS-physical function and self-image, and patients with curves ≥ 45° had reduced self-image.

**Conclusion:**

Long-term results were satisfactory in most braced patients and similar in late-onset juvenile and idiopathic adolescent scoliosis.

## Background

A recent Cochrane systematic review evaluated the efficacy of bracing in adolescent idiopathic scoliosis (AIS) [1]. The full text of 128 studies was reviewed, but only one randomized controlled trial [2] and one prospective cohort study [3] could be included. From the results of these studies it was concluded that the evidence in favour of using braces is very low quality, making generalization difficult [1]. The authors recommended that future research should focus on short- and long-term patient-centred outcome. Unfortunately, prospective studies that started > 20 years ago did not use a randomized design, which limits their ability to evaluate causal relationships between bracing, curve progression, and Health Related Quality of Life (HRQL). The most comprehensive studies on long-term outcome including HRQL measures have been published by Danielsson and Nachemson and present outcome 22 years after treatment [4-7]. In a prospective study we recently reported the results in 109 patients treated with Boston brace at an average of 19.2 years earlier [8]. We found that the major curve size was not different from the pre-brace curve. Eighty percent worked full time, 88% had delivered a baby, and the average HRQL-scores were comparable with the results reported by Danielsson and Nachemson and in the same range as in the normal population. Scoliosis Research Society - 22 (SRS - 22) scores were in the same range as in a study evaluating 109 patients 10-years after surgery using third-generation instrumentation [9]. In particular, the mean score for self-image was identical, while patient satisfaction was slightly worse.

Weigert et al. compared results in 49 patients with Boston brace only, 41 patients with surgery, and 33 patients treated with brace and surgery at minimum 2 years follow-up [10]. They reported that surgically treated patients had better scores for post-treatment self-image and satisfaction, while the brace only treated patients had a higher level of general activity. They also found that the double-treated group scored highest in most of the domains including satisfaction, and concluded that brace treatment did not have any long-lasting negative effect on HRQL, causing no more psychological harm to the teenager than providing benefit.

The aim of the present study was to evaluate progression of the scoliotic curve and to report HRQL, using both validated scoliosis specific and generic questionnaires, in patients with idiopathic scoliosis 16 years and more after Boston brace treatment. We particularly wanted to compare results in patients presenting at an early age (late-onset juvenile scoliosis, 7-9 years) with patients presenting after 10 years (AIS), and in patients with curve progression to ≥ 45° and < 45°, respectively, with the results in those who had surgery after bracing.

## Methods

Long-term results in 109 of 138 patients with AIS who were treated with Boston brace at Sophies Minde Orthopaedic Hospital (Orthopaedic Department, Oslo University Hospital, Rikshospitalet) in Oslo, Norway from 1976-88 and who had their last earlier follow-up no longer than 2 years after brace weaning, have been published previously [8]. These patients were not included in the present study.

We carefully evaluated the medical journals of the remaining 480 patients who had been braced for scoliosis and prospectively followed during the same period, and identified 369 patients with idiopathic scoliosis. These patients were invited for long-term follow-up. We included both patients with late-onset juvenile and AIS. Late-onset juvenile idiopathic scoliosis is discovered at 7 to 10 years, while AIS is discovered at 10 years of age or older [11].

In general we followed the SRS committee criteria, which recommend bracing for curves that measure between 25° and 40° and surgery for curves that are greater than 45° during the growth phase and that the brace should be prescribed until growth of the spine has stopped http://www.srs.org/professionals/conditions_and_treatment/adolsescent_idiopathic_scoliosis/treatment.htm. Accordingly, the indication for bracing was a major scoliotic curve ≥ 20° with an observed progression > 5° after 4 months and Risser sign < 4. Prior to bracing standing radiographs were taken in the front and lateral projections. Patients had follow-up with clinical and radiological examination at 4 months intervals throughout the brace treatment period. Wearing of the brace was assessed by one orthopaedic surgeon (JEL) and reported as used as prescribed, irregular, or aborted. Patients were recommended to use the brace for 23 hours daily. Wearing of the brace < 20 hours daily was described as irregular.

Physiotherapy was not prescribed in addition to bracing, but in general patients were recommended to participate in physical activity at school and leisure time. They were advised not to carry heavy loads, and in case they had a long walk to school they were given two sets of schoolbooks.

Brace weaning was carried out either 2 years after menarche or at Risser sign 5, in some patients at Risser sign 4. After weaning all patients had follow-up examination at 6, 12, and 24 months.

A standardised form was used to obtain clinical and radiological data. Radiological measurements were performed by an orthopaedic surgeon (JEL) and controlled by an experienced radiologist (RG). Both used the Cobb method, digital measurements were used at long-term follow-up. The intra-observer error for the Cobb angle was about 3° in a recent study using manual and digital measurements, and < 5° in a previous study [12,13]. In the present study the measurement error was within these limits as evaluated by the reproducibility of radiographic readings of repeated measurements of all radiographs from 10 patients at regular intervals. In patients with double thoraco-lumbar curves the largest curve prior to bracing was defined as the major curve.

### Questionnaires

At long-term follow-up, all patients first filled in a standardised questionnaire and thereafter they had clinical and radiological examination. The questionnaire comprised validated measures of pain, disability, quality of life and work, and questions about demographics.

Evaluation of work status included questions about paid work (full-time, part-time, not working) and status if not working (on sick leave, vocational or medical rehabilitation, disability pension, unemployed, homemaker, or student) [14]. Norway has a National Social Security System that covers all inhabitants. Patients on sickness certification receive 100% benefit up to one year. Thereafter, they receive medical or vocational rehabilitation in order to reduce disability. If the patients are not able to work after rehabilitation they receive a disability pension with a lower benefit.

The General Function Score (GFS) was used to measure back-related disability in activities of daily living [15]. Patients answered nine questions wherein 100% represents maximum disability.

The Norwegian version of the original Oswestry Disability Index (ODI) (version 1.0) was used to evaluate back-specific disability [14,16]. This score has 10 questions about pain and pain-related disability in activities of daily living and social participation wherein 100% represents the worst imaginable pain and disability.

Patients rated their overall function by the Global Back Disability Question [14]. This is a single question designed to measure the patients' overall rating of their back disability today. There were five response alternatives ranging from "excellent, none or unimportant complaints," to "miserable, worse, not self-reliant in activities of daily living".

EuroQol is a generic (non-disease specific) instrument for measurement of quality of life [17-19]. The questionnaire includes five items regarding quality of daily life, covering the domains of mobility, self-care, usual activities, pain and discomfort, and anxiety and depression (EQ-5D), and a visual analogue score for assessment of overall current health (EQ-VAS). The index score (EQ-5D) range from -0.59 for the worst possible health state to +1.00 for the best possible health state. Patients rate their overall current health (EQ-VAS) from 0 (worst imaginable) to 100 (best imaginable).

The Scoliosis Research Society 22 questionnaire (SRS-22) is validated and widely used for evaluation of health-related quality of life in AIS [20,21]. A recently translated and validated Norwegian version was used in the present study [22]. The SRS-22 covers five domains (function/activity, pain, self-perceived image, mental health, and satisfaction with treatment). Each item has 5 verbal response alternatives ranging from 1 (worst) to 5 (best). Results are expressed as the mean for each domain ranging from 1 (worst) to 5 (best).

### Ethics

The committee for medical research ethics in the health Region South-East in Norway and the institutional review board (hospital's patient ombudsman) approved the study (REK 2010-3677).

### Statistical analysis

All patients with available data were included. Statistical analyses were performed with SPSS software, version 18.0 (SPSS Inc., Chicago) and Statistical Analysis System (SAS version 9.2; Cary, NC). Results are presented as means (standard deviation, range) or percentages. The normal distribution of baseline, follow-up data, and differences were checked by histograms. The success rate at maturity was calculated according to Nachemson and Peterson [3]. They defined success of treatment as an increase in the primary curve of less than 6° from the start of bracing. Surgical patients were classified as non-success. In addition we calculated the percentage of patients who had a decrease ≥ 6°. Baseline characteristics in those who did not attend and those who attended long-term follow-up and in patients with adolescent and late-onset juvenile idiopathic scoliosis, respectively, were compared with independent t-tests. A General Linear Model One-way analysis of variance was used to test differences in continuous variables at baseline, weaning, and long-term between the 3 subgroups: 1) brace treated patients with final major curve < 45°, 2) brace and surgery, and 3) brace and final major curve ≥ 45°, and between patients with different curve types. With the assumption of unequal variances in unequally sized groups, Dunnett's T3 was used for post hoc multiple comparisons. Chi-square analyses were applied for testing of categorical variables. Spearman-R test was used to evaluate the correlation between pre-brace curve size and follow-up curve size and HRQL.

## Results

### Patients and pre-brace scoliotic curves

Nine patients with early-onset juvenile (4 to 6 years) were excluded from analyses.

272 (76%) of 360 patients, 251 (92%) women, filled in the questionnaire, and had additional clinical and radiological examination at follow-up at mean 24.7 (range 16 to 32) years after Boston brace treatment. The mean age was 40.4 (31 to 48) years at follow-up.

There were fifty-eight (21%) patients with late-onset juvenile (7 to 9 years) and 214 patients with adolescent idiopathic scoliosis. The curve type was thoracic in 189 (70%), thoraco-lumbar in 55 (30%) and lumbar in 28 (10%) patients.

Mean (standard deviation) age at start of bracing was 13.1 (1.9) years, bone age 12.5 (1.9) years, and age at menarche 13.4 (1.2) years. The mean primary curve was 33.0 (20 to 57)°, 28.4 (1 to 58)° at brace weaning, and 32.5 (7 to 80)° at follow-up.

Patients not attending follow-up (n = 88) were not different from those attending, except that their major curve at weaning was smaller (25.4 (6 to 53)°, p = 0.02) (Table 1).

**Table 1:** Baseline characteristics in 272 Boston braced patients

Characteristic	Had long-term follow-up n = 272	Did not attend long-term follow-up n = 88	p-value
Age at start brace treatment (years)	13.1 (1.9)	12.9 (2.3)	0.83
Bone age at start brace treatment (years)	12.5 (1.9)	12.3 (2.4)	0.67
Age at menarche (years)	13.4 (1.2)	13.3 (1.4)	0.61
Age at weaning (years)	15.8 (1.5)	15.5 (1.7)	0.35
Bone age at weaning (years)	15.1 (1.3)	14.7 (1.7)	0.20
Major curve at start brace treatment (°)	33.0 (7.0)	32.4 (8.0)	0.41
Major curve at weaning (°)	28.4 (10.4)	25.4 (9.7)	0.02
Age at operation (years)	15.8 (1.9)	16.1 (1.4)	0.80

Values are means (standard deviation).

### Pre-brace characteristics and major curve development of late-onset juvenile and adolescent idiopathic scoliosis

**Figure 1:**
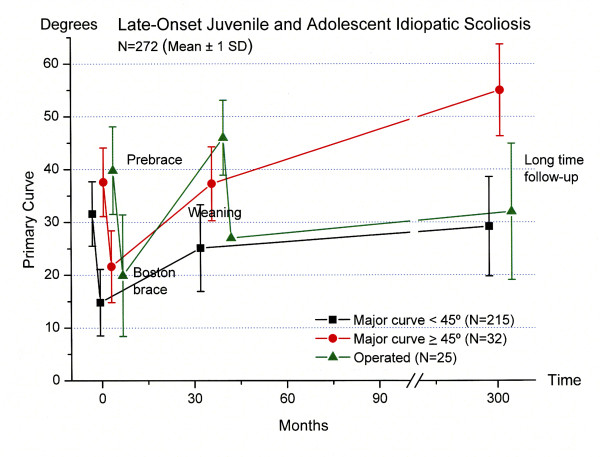
**Longitudinal development of the major curve in late-onset juvenile (N = 58) and adolescent idiopathic scoliosis (N = 214)**. Mean Cobb angle ± 1 SD prebrace, at brace weaning, and at long-term follow-up in 272 patients.

The characteristics of patients with late-onset juvenile and AIS are presented in Table 2 and Figure 1. The mean age of 10.7 (7.5 to 14.8) years at start bracing of patients with late-onset juvenile idiopathic scoliosis, was significantly (p < 0.001) lower than the age of patients with AIS (13.8 (10.7 to 17.0) years). The corresponding values for estimated maturation by skeletal age assessments at start of treatment were 10.5 (2.0) and 13.1 (1.4) (p < 0.001). The late-onset juvenile versus adolescent patients stopped bracing significantly (p < 0.001) earlier (Table 2), but the major curves in patients with late-onset juvenile and AIS were not different, neither at start bracing, weaning nor at long-time follow-up (Figure 1).

**Table 2:** Baseline characteristics in 272 Boston braced patients with late-onset juvenile or adolescent idiopathic scoliosis at mean follow-up of 24.7 years

Characteristic	Adolescent n = 214	Late-onset n = 58	p-value
Age at start of brace treatment (years)	13.8 (1.3)	10.7 (1.9)	< 0.001
Bone age at start brace treatment (years)	13.1 (1.4)	10.5 (2.0)	< 0.001
Age at menarche (years)	13.2 (1.1)	13.2 (1.1)	0.22
Age at weaning (years)	16.1 (1.1)	14.5 (1.8)	< 0.001
Bone age at weaning (years)	15.4 (1.0)	14.4 (1.7)	< 0.001
Major curve at start brace treatment (°)	33.3 (6.9)	31.9 (7.1)	0.18
Major curve at weaning (°)	29.0 (10.0)	26.3 (11.8)	0.09
Age at operation (years)	15.4 (1.8)	14.4 (1.9)	0.22
Major curve at long-time follow-up (°)	33.0 (12.7)	30.4 (12.9)	0.17

Values are means (standard deviation).

### Pre-brace characteristics and major curve development in patients treated with brace, brace and surgery, and in brace treated patients with curve size ≥ 45°

The primary scoliotic curves were significantly (p < 0.001) larger at start bracing and at weaning in the later operated patients, with a mean value of 39.8 (28 to 57)° and 46.0 (34 to 58)°, respectively (Table 3). In braced patients who had a major curve *≥ *45° at follow-up, the mean primary curves of 37.6 (21 to 48)° at baseline and 37.3 (10 to 46)° at weaning were significantly (p < 0.001) increased compared to the cohort mean, and at start bracing the primary curve was not different from the subgroup which later had surgery.

**Table 3:** Baseline characteristics in 272 Boston braced patients with late-onset juvenile or adolescent idiopathic scoliosis at mean follow-up of 24.7 years

Characteristic	Brace n = 215	Brace and surgery n = 25	Brace and major curve ≥ 45° at follow-up n = 32
Age at start of brace treatment (years)	13.2 (1.8)	11.4 (2.3)***	13.8 (1.4)
Bone age at start brace treatment (years)	12.7 (1.8)	10.7 (2.1)***	12.9 (1.1)
Age at menarche (years)	13.3 (1.2)	13.8 (1.0)	13.9 (1.2)*
Age at weaning (years)	15.8 (1.4)	14.4 (1.6)***	16.6 (1.5)**
Bone age at weaning (years)	15.3 (1.1)	13.9 (1.8)***	15.4 (1.2)
Major curve at start brace treatment (°)	31.6 (6.1)***	39.8 (8.3)	37.6 (6.5)
Major curve at weaning (°)	25.1 (8.2)***	46.0 (7.1)***	37.3 (7.0)***
Age at operation (years)		15.8 (2.8)	
Major curve at long-time follow-up (°)	29.2 (9.4)	32.0 (12.9)	55.0 (8.7)***

Values are means (standard deviation).

*** Significantly different from the other 2 groups (p < 0.001)

** Significantly different from the Brace group (p < 0.005)

* Significantly different from the Brace group (p < 0.05)

**Figure 2:**
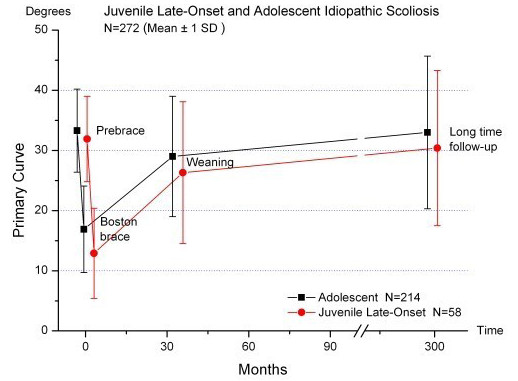
**Longitudinal development of the major curve in late-onset juvenile and adolescent idiopathic scoliosis classified according to the status at follow-up**. Mean Cobb angle ± 1 SD prebrace, at brace weaning, and at long-term follow-up in patients with final major curve < 45° (N = 215), final major curve ≥ 45° (N = 32), and operated patients (N = 25).

The major curve development is shown in Figure 2. Thirty-five patients, 32 (13%) of the 247 treated with brace, and in addition 3 (12%) of the 25 treated with brace and surgery had a major curve of ≥ 45° at follow-up. The curve exceeded 60° in 9 patients, including 2 who had surgery. The success rate according to curve size progression of < 6° was 89% at weaning and 69% at long-term, while seventy-two (26%) patients had a decrease in curve size ≥ 6° (range 6-24)°.

Surgical correction after bracing was performed in twenty-five (9%) patients. Eighteen were operated within one year after weaning. Thirteen had Harrington instrumentation, 8 had Cotrel-Dubousset, and 4 had Harry-Luque. Four patients were re-operated, while one patient had two re-operations. The surgically treated patients were significantly (p < 0.001) younger both chronologically and in maturation at the start of bracing, with a mean age of 11.4 (2.3) and 10.7 (2.1) years, respectively (Table 3).

### Socio-demographics and Health Related Quality of Life at follow-up

**Figure 3:**
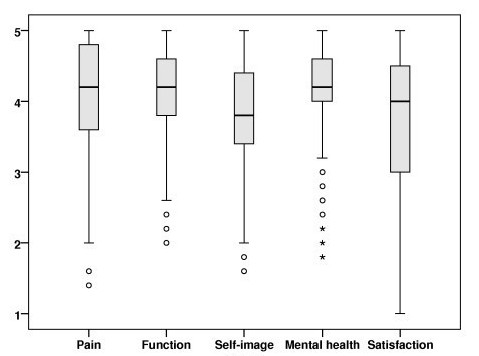
**Quality of life at long-term follow-up in late-onset juvenile and adolescent idiopathic scoliosis treated with the Boston brace**. Box-plot showing median with 25- and 75 percentiles and outliers for each domain of the SRS-22 (1 = worst possible, 5 = best possible).

Work status was full-time in 76% and part-time in 10%. Full-time disability pension was taken by 6%, rehabilitation by 1%, sick leave by 2%, while 2% were students or homemakers, respectively. 87% had delivered a baby, 50% had back pain in pregnancy. Twenty-eight percent considered their back as excellent, 41% good, 26% fair, 5% poor (including 1 patient who considered his back as miserable). The mean (standard deviation) GFS was 7.4 (10.8), ODI 9.3 (11.0), EQ-5D 0.82 (0.2), EQ-VAS 77.6 (17.8), SRS-22: pain 4.1 (0.8), mental health 4.1 (0.6), self-image 3.7 (0.7), function 4.0 (0.6), satisfaction with treatment 3.7 (1.0). Figure 3 shows a box-plot of SRS-scores.

We did not find a difference in socio-demographic characteristics and HRQL-scores at long-term of patients with late-onset juvenile and adolescent scoliosis.

**Figure 4:**
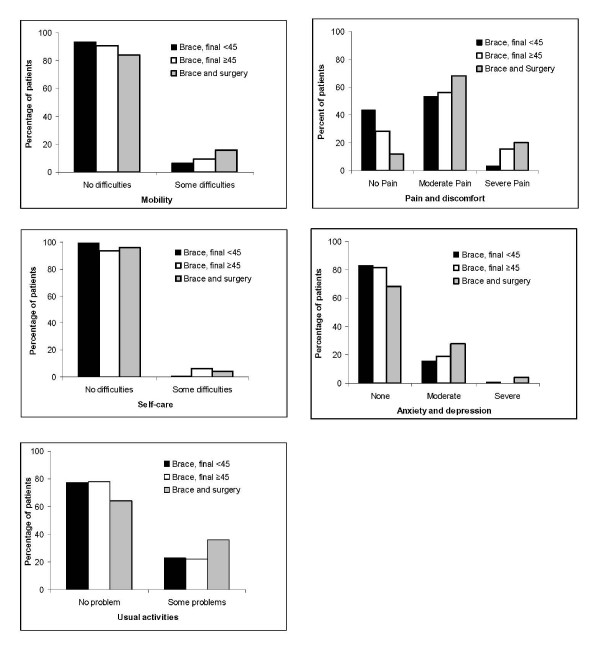
**Quality of life at long-term follow-up in late-onset juvenile and adolescent idiopathic scoliosis treated with the Boston brace**. Percentages of patients are given for each domain (mobility, pain and discomfort, usual activities, self-care, and anxiety and depression) in patients with brace and final curve < 45°, brace and final curve ≥ 45°, and brace and surgery. Significantly (p < 0.01) more patients in the two latter groups reported moderate and severe pain.

Results in patients without and with surgery and braced only patients with curves ≥ 45 ° are presented in Table 4. Significantly (p < 0.05) more surgical patients were smoking and had changed their job compared with the other groups, while fewer patients with a large major curve at long-term were married (Table 4). Results for ODI, EQ-5D, EQ-VAS, and the SRS-22 domains physical function and self-image were significantly (p < 0.05) worse in surgical patients, compared with a large major curve, Table 5. Self-image was significantly worse in braced only patients with curve size ≥ 45° compared with those with curve size < 45°. The five domains of EuroQol are shown in Figure 4. Significantly (p < 0.01) more patients with a major curve ≥ 45° or surgery reported moderate and severe pain.

**Table 4:** Socio-demographic characteristics in 272 Boston braced patients with late-onset juvenile or adolescent idiopathic scoliosis at mean follow-up of 24.7 years

Characteristic	Brace only N = 215	Brace and surgery N = 25	Brace and major curve ≥ 45° at follow-up n = 32
Educational level			
Primary school (9 year)	6	9	7
High school (12 year)	18	26	32
University college	76	66	61
Work status	78	64	72
Working full time	9	16	9
Working part- time	2	4	0
Student	2	4	0
Homemaker	2	0	0
On sick leave	1	4	4
Rehabilitation	6	8	15
Disability pension			
Changed job because of back pain or disability	26	46*¤	27
Scoliosis influenced my choice of	29	42	44
education and job	32	33	48
Comorbidity	17	42*¤	19
Smoking	84	72	63#
Married/living together			
Born children (n = 259)	87	91	86
Pain in pregnancy (n = 225)	50	53	50

Percentages are given.

Brace compared with brace and surgery: * p < 0.05

Brace compared with brace and major curve ≥ 45°: # p < 0.05

Brace and surgery compared with brace and major curve ≥ 45°:¤ p < 0.05

**Table 5:** Results in 272 Boston braced patients with late-onset juvenile or adolescent idiopathic scoliosis at mean follow-up of 24.7 years

Outcome	Brace n = 215	Brace and surgery n = 25	Brace and major curve ≥ 45° at follow-up n = 32
Global Back Question	32	4	22
Excellent	41	56	34
Good	23	36	34
Fair	5	4	6
Poor			
General Function Score (0-100)	6.4 (10.3)	12.2 (14.1)	11.1 (17.5)
Oswestry Disability Index			
(0-100)	8.0 (10.8)	17.0 (15.0)*	12.6 (15.3)
EQ - 5D (-0.5 to 1.0)	0.83 (0.19)	0.66 (0.31)*	0.73 (0.27)
EQ - VAS (0-100)	80.0 (16.6)	69.2 (18.4)*	72.0 (21.4)
SRS-22 (1-5)			
Pain	4.1 (0.8)	3.6 (1.0)	4.0 (0.8)
Physical function	4.1 (0.6)	3.6 (0.8)*	3.9 (0.9)
Mental health	4.1 (0.6)	3.9 (0.7)	4.2 (0.7)
Self -image	3.8 (0.7)	3.4 (0.8)*	3.3 (0.8)##
Satisfaction	3.7 (1.0)	3.8 (0.7)	3.3 (1.1)

Percentages or means (standard deviations) are given.

Brace compared with brace and surgery: * p < 0.05

Brace compared with brace and major curve ≥ 45°: ## p < 0.01

We did not find a difference in HRQL-scores in patients with a thoracic, thoraco-lumbar, or lumbar major curve. Results in males were not different from females. The major pre-brace curve correlated (Spearman R) moderately with the final major curve (r = 0.46), while none of the correlations between the major pre-brace curve and the SRS-domains reached r > 0.10. The SRS-domains were better correlated: pain and function (r = 0.79); self-image and function (r = 0.71); self-image and pain (r = 0.67); and mental health and function (r = 0.57); self-image and satisfaction (r = 0.42); pain and mental health (r = 0.39); pain and satisfaction (r = 0.37), except mental health and satisfaction (r = 0.12).

## Discussion

Results at mean 24.7 years after treatment with Boston brace for AIS are in agreement with previous studies on bracing [8,23]. Curve progression was < 6° in 69% of the patients and the average major curve size was almost identical to the pre-brace curve. The percentage that had later surgery was in the lower range compared with a recently published systematic review reporting that the percentage of brace treated patients with later surgery ranges from 1% to 43% [24]. HRQL was slightly worse in operated patients, but these patients had larger curves at weaning. About half of them were operated with Harrington instrumentation, but any inference about the results of current methods of surgery for idiopathic scoliosis cannot be made from the results of the present study. Our results are slightly different from Weigert et al. [10], who reported better post-treatment self-image and satisfaction in surgical patients, but results are not directly comparable because most patients had Cotrel-Dubousset instrumentation and follow-up was much shorter. However, previous studies have reported that HRQL at long-term follow-up after Harrington instrumentation for AIS is comparable with the normal population [25,26].

Except for self-image and the pain domain on EuroQol the reported HRQL was not related to curve size at follow-up. Some of the patients with large curves at follow-up had refused surgery, but most braced only patients had a curve size < 45° at maturity with an average of 37.3°.

Results suggest that average curve progression and HRQL are satisfactory at long-term, although a higher percentage of patients report moderate and severe pain and discomfort and difficulties with usual activities as compared with 40-49 year old women in the United Kingdom national questionnaire survey using the EQ-5D questionnaire [27]. They reported that the population percentage reporting any problem in this age and gender category was 11% for mobility, 4% for self-care, 12% for usual activity, 27.5% for pain and discomfort, and 21% for anxiety and depression. Figure 4 shows that from 60 to 90% of the patients in the present study reported to have either moderate or severe pain. Although the average SRS-22 pain score was low and that the three response alternatives for EQ pain and discomfort do not allow for reporting some or occasional pain, the present study indicates that the majority of patients with idiopathic scoliosis are not pain free at long-term. Despite some pain, most patients have little functional limitations and are working.

The design of the present study does not allow for causal inference. With the lack of control group and a randomized design we cannot exclude that curve development and HRQL would have been in the same range without bracing. The results of one prospective study that included a control group and one randomised trial that compared soft and rigid braces suggest an advantage of bracing on curve progression [3,28]. Nevertheless, based on these studies, a recent Cohrane systematic review concluded that there is very low evidence in favour of using braces [1]. Results from ongoing randomized controlled trials are expected to improve the knowledge in this field [29,30]. Because both bracing and surgery is demanding for the adolescent girl, the long-term prognosis in untreated patients must be considered including curve progression, HRQL, and costs. Recently, Danielsson et al. compared long-term outcome after bracing and observation only [31]. They found no difference in curve progression and long-term HRQL, but the cohorts were different at baseline regarding the number of patients who had an observed curve progression of > 5°. According to their conclusion many patients with idiopathic scoliosis are over-treated, and if bracing is limited to patients with documented curve progression the number who are possibly over-treated will be reduced.

Our results are in agreement with Aulisa et al. reporting that conservative treatment does not severely impact on HRQL in AIS patients [32]. However, results are not directly comparable because they included much younger patients (mean age 15.4 years at follow-up) and applied different braces. Thus, they evaluated the effects of brace treatment in young adolescents while the patients in the present study were middle aged. We cannot exclude that bracing had negative impact on some patients, and the scores for satisfaction with treatment suggest that bracing was demanding, but we did not find a negative impact on mental health at long-term. In contrast with the results in the current study, Aulisa et al. reported better results in boys.

The percentage reporting pain during pregnancy is comparable to women without AIS and in agreement with the results of a large previously published case-control study [5]. Questions about pregnancy and delivery are often raised in AIS. In agreement with previous studies our results indicate that patients can be reassured that scoliosis does not affect pregnancy or delivery.

A meta-analyses concluded that bracing is effective, but slightly less effective in patients with juvenile onset [33]. The present study did not include patients with early-juvenile onset, but there was no difference in curve development during bracing or at long-term or in HRQL at long-term between patients with late-juvenile and adolescent onset. Thus, similar results should be expected in these groups.

## Conclusion

We conclude that long-term results were satisfactory in most of 272 patients with late-onset juvenile and AIS treated with Boston brace in average 24.7 years earlier. Nine percent had surgery and 13% had curve progression to ≥ 45° at follow-up. HRQL was slightly decreased in these patients, SRS-22 scores for self-image was significantly lower in both groups. Self-report indicates that future patients can be reassured that scoliosis does not affect pregnancy and delivery, and that most patients are expected to work and have HRQL in the normal range at long-term.

## Funding

The study was supported by grants from the Sophies Minde AS Foundation.

## Competing interests

The authors declare that they have no competing interests.

## Authors' contributions

JEL designed the study, collected all the data at baseline, and contributed to collection and interpretation of data at long-term follow up as well as manuscript drafting. HS contributed to the design of the study, collection and interpretation of data, and carried out the data analyses and manuscript drafting. RG carried out all the radiological measurements at long-term follow-up. JIB contributed to the design of the long-term follow-up, collection and interpretation of data, carried out the data analyses, and wrote the manuscript. All authors read and approved the final manuscript.
